# Gi Protein Modulation of the Potassium Channel TASK-2 Mediates Vesicle Osmotic Swelling to Facilitate the Fusion of Aquaporin-2 Water Channel Containing Vesicles

**DOI:** 10.3390/cells7120276

**Published:** 2018-12-19

**Authors:** Mariangela Centrone, Maria Penelope De Santo, Isabella Nicotera, Cristina Labate, Marianna Ranieri, Annarita Di Mise, Maria Grazia Mola, Maria Mastrodonato, Rosangela Elliani, Riccardo Barberi, Vincenzo Formoso, Grazia Tamma, Giovanna Valenti

**Affiliations:** 1 Department of Biosciences, Biotechnologies and Biopharmaceutics, University of Bari Aldo Moro, Bari 70125, Italy; mariangela.centrone@uniba.it (M.C.); marianna.ranieri@uniba.it (M.R.); annarita.dimise@uniba.it (A.D.M.); mariagrazia.mola@uniba.it (M.G.M.); grazia.tamma@uniba.it (G.T.); 2 Physics Department, University of Calabria, Rende 87036, Italy; maria.desanto@fis.unical.it (M.P.D.S.); cristina.labate87@gmail.com (C.L.); riccardo.barberi@fis.unical.it (R.B.); 3 Department of Chemistry and Chemical Technologies, University of Calabria, Rende 87036, Italy; isabella.nicotera@unical.it (I.N.); rosangela.elliani@hotmail.it (R.E.); 4 Department of Biology, University of Bari Aldo Moro, Bari 70125, Italy; maria.mastrodonato@uniba.it; 5 Institute of Nanotechnology-CNR (CNR-Nanotec), Cosenza Unit, Rende 87036, Italy; 6 Sistema Tecnologico MaTeRIA, University of Calabria, Rende 87036, Italy; 7 Interuniversitary Consortium “Istituto Nazionale Biostrutture e Biosistemi” (INBB), Rome 00136, Italy; 8 Center of Excellence in Comparative Genomics (CEGBA), University of Bari Aldo Moro, Bari 70125, Italy

**Keywords:** Gi protein, TASK-2, Aquaporin-2, exocytosis, swelling

## Abstract

Vesicle fusion is a fundamental cell biological process similar from yeasts to humans. For secretory vesicles, swelling is considered a step required for the expulsion of intravesicular content. Here this concept is revisited providing evidence that it may instead represent a general mechanism. We report the first example that non-secretory vesicles, committed to insert the Aquaporin-2 water channel into the plasma membrane, swell and this phenomenon is required for fusion to plasma membrane. Through an interdisciplinary approach, using atomic force microscope (AFM), a fluorescence-based assay of vesicle volume changes and NMR spectroscopy to measure water self-diffusion coefficient, we provide evidence that Gi protein modulation of potassium channel TASK-2 localized in AQP2 vesicles, is required for vesicle swelling. Estimated intravesicular K^+^ concentration in AQP2 vesicles, as measured by inductively coupled plasma mass spectrometry, was 5.3 mM, demonstrating the existence of an inwardly K^+^ chemical gradient likely generating an osmotic gradient causing vesicle swelling upon TASK-2 gating. Of note, abrogation of K^+^ gradient significantly impaired fusion between vesicles and plasma membrane. We conclude that vesicle swelling is a potentially important prerequisite for vesicle fusion to the plasma membrane and may be required also for other non-secretory vesicles, depicting a general mechanism for vesicle fusion.

## 1. Introduction

Vesicle fusion is a critical event in the life of every cell: protein sorting, membrane assembly, mitosis, ER/Golgi trafficking and secretion depend upon it. It is the basis of hormone and neurotransmitter release, though there is not yet a deep understanding of the functional pathway of vesicle fusion events at the level of physical forces involved in the mechanism of release. 

It has been suggested that vesicle swelling is involved in the exocytotic process [1,2,3]. In secretory vesicles, a specific set of ion channels expressed in the membrane has been proposed to contribute to the secretory granule swelling [4,5,6,7,8]. In isolated zymogen granules from exocrine pancreas and parotid glands, Cl_2_ and K^+^ ion channels have been found expressed and shown to be involved in swelling [9]. Gαi3 protein has been suggested as major regulator of the swelling process since it is implicated in the regulation of both channels [6,10,11,12,13,14,15,16,17,18]. Similarly, Go protein has been identified at the synaptic vesicle membrane and is involved in swelling induced by active mastoparan—an amphiphilic tetradecapeptide from wasp venom—which has been demonstrated to activate the GTPase activity of Go/i proteins [19,20,21]. 

During the secretory process, the general assumption is that increase in vesicle volume is required for the expulsion of intravesicular content. Vesicle swelling implies rapid water entry into the vesicle, likely triggered by a local osmotic gradient. Worthy of interest, members of the aquaporin water channel family have been identified in zymogen granules in rat pancreatic acinar cells (AQP1) [4], in secretory granules in rat parotid acinar cells (AQP5) [22] in synaptic vesicles from rat brain (AQP1 and AQP6) [5] and in intracellular vesicles in rat kidney (AQP6) [23]. These observations suggest that AQPs in secretory granules and vesicles may mediate their volume regulation [24]. The proposed hypothesis for regulation of secretory vesicle swelling and fusion to the plasma membrane implies that the Gi protein expressed on the vesicle membrane activates associated Cl_2_ and K^+^ channels causing ion entry, thus creating an osmotic gradient resulting in AQP-mediated water flow into the vesicle lumen resulting in vesicle swelling and subsequent fusion at the plasma membrane [6]. 

Among the known aquaporins, the vasopressin-sensitive AQP2 is also expressed in intracellular vesicles that traffic to the apical membrane in response to vasopressin stimulation a process essential to maintain water homeostasis. Specifically, in renal collecting duct principal cells, the primary cellular targets of vasopressin are V2 receptors coupled to adenylyl cyclase via the cholera toxin-sensitive G protein Gs; activation of this pathway leads to an increase in intracellular cAMP levels and activation of cAMP-dependent protein kinase. The signalling cascade activated by vasopressin includes AQP2 phosphorylation, intracellular calcium mobilization and RhoA dependent actin depolymerization, playing a key role in the translocation of AQP2 bearing vesicles and insertion of the AQP2 water channel into the apical membrane [25,26,27].

While AQP2 vesicles do not secrete their content our previous study demonstrated that, as for secretory vesicles, a member of the Gi family, Gαi3, is co-expressed with AQP2 in subapical vesicles and is required for fusion [28]. In fact, treatment of collecting duct cells with pertussis toxin (PTX) which ADP-ribosylates and thereby uncouples G proteins of the Gi and Go types from their cognate receptors, inhibited both the vasopressin-induced increase in water permeability and the redistribution of AQP2 from an intracellular compartment to the apical membrane [28]. This finding suggest the hypothesis that Gαi3 might regulate an ion channel gating promoting osmotic AQP2 vesicle swelling and fusion to the plasma membrane. 

The present study was therefore undertaken to address the question of whether vesicle swelling is also a prerequisite for fusion of AQP2 vesicles committed to insert a channel into the plasma membrane.

## 2. Materials and Methods

### 2.1. Materials

Calcein green-AM was obtained from Life Technologies (Monza, Italy). 4,4′-Diisothiocyanatostilbene-2,2′-disulfonic acid disodium salt (DIDS), quinidine, mercury(II) chloride, desmopressin (dDAVP) and Protein A-Sepharose^®^ from Staphylococcus aureus were purchased from Sigma (Sigma-Aldrich, Milan, Italy). Forskolin was obtained from Fermentek Biotechnology (Jerusalem, Israel). Pertussis Toxin was purchased from Enzo Life Sciences Biotechnology (Farmingdale, NY, USA). Biocytin Hydrazide and streptavidin beads were purchased from EZ-Link^®^ Pierce (Rockford, IL, USA).

### 2.2. Antibodies

AQP2 was detected using a specific antibody (C-tail Ab) raised against a synthetic peptide corresponding to the last 15 C-terminal amino-acids of human AQP2 [29]. Monoclonal Gαi3 antibody recognizing amino-acid 339–354 at the C-terminus was purchased from Santa Cruz Biotechnology (Dallas, TX, USA). Anti-Potassium Channel TASK-2 (Kcnk5) was bought from Sigma. Anti-Na^+^/K^+^-ATPase subunit alpha 1 (clone C464.6) was from Millipore (Milano, Italy). Antibody anti-ClC-K (#ACL004) was from Alomone Labs (Jerusalem, Israel). Antibody against VAMP2 was a kind gift from Professor J.E. Pessin [30]. Secondary goat-anti-rabbit-IgG and anti-mouse-IgG antibodies conjugated to Alexa Fluor 488 were from Molecular Probes, Eugene, OR, USA.

### 2.3. Cell Culture

Mouse Cortical Collecting Duct cells (MCD4), stably expressing human AQP2, generated as described elsewhere [31], were grown in Dulbecco’s modified Eagle’s medium (DMEM/F12) supplemented with 5% fetal bovine serum, 2 mM l-glutamine, 100 i.u./mL penicillin, 100 µg/mL streptomycin and 5 µM dexamethasone at 37 °C in 5% CO_2_. 

### 2.4. Preparation of Membrane Vesicles

Vesicles were prepared from rat kidney papillae or MCD4 cells. The inner medulla from rat kidney slices were excised and cut in small pieces. These pieces or MCD4 cells were homogenized manually with a mini-potter in ice-cold Isolation medium (220 mM mannitol, 70 mM sucrose, 5 mM EGTA, 1 mM EDTA, 20 mM Tris-HCl pH 7.4). Nuclei and mitochondria enriched fractions were removed by centrifugation at 8000× *g* for 20 min while membrane was removed by centrifugation at 17,000× *g* for 1 h. The supernatant was spun at 200,000× *g* in a Beckman Rotor TLA 120.1 for 1 h at 4 °C. The final pellet, enriched in intracellular vesicles, was gently resuspended in Isolation medium using a 30-gauge needle and used for experiments.

### 2.5. Gel Electrophoresis and Western Blotting

Proteins were separated on 10% or 13% bis-tris acrylamide gels under reducing conditions. Protein bands were electrophoretically transferred onto Immobilon-P membranes (Millipore Corporate Headquarters, Billerica, MA, USA) for Western blot analysis, blocked in TBS-Tween-20 containing 3% BSA and incubated with primary antibodies overnight. Immunoreactive bands were detected with secondary antibody conjugated to horseradish peroxidase (HRP) obtained from Santa Cruz Biotechnologies (Tebu Bio, Milan, Italy). Membranes were developed using SuperSignal West Pico Chemiluminescent Substrate (Pierce, Rockford, IL, USA) with Chemidoc System (Bio-Rad Laboratories, Milan, Italy). Representative figures are shown. Densitometry analysis was performed with Scion Image. Data are summarized in histograms with GraphPad Prism (Graphpad Software Inc. La Jolla, CA, USA).

### 2.6. Immunofluorescence

MCD4 cells were grown on polyester Transwell inserts and incubated in the absence or in the presence of PTX (2 µg/mL) for 3 h at 37 °C and either stimulated with 100 µM forskolin for 30 min or left under basal conditions and then fixed using 4% paraformaldehyde in phosphate-buffered saline (PBS). To test the effect of quinidine, in a set of experiments cells were preincubated with 10 µM quinidine for 45 min before stimulation with forskolin. Cells were permeabilized with 0.1% Triton X-100 in PBS for 5 min and nonspecific binding sites were blocked with 1% bovine serum albumin in PBS at room temperature for 1 h. Cells were then incubated with specific antibodies for 2 h at 37 °C. After washing in PBS, cells were incubated with the appropriate fluorescent secondary antibodies for 30 min at room temperature, washed in PBS and mounted on glass slides with Mowiol. 

For protein localization in renal tissue, rat kidneys were fixed by immersion in 4% paraformaldehyde in PBS at 4 °C overnight, cryopreserved in 30% sucrose in PBS for 12 h and then embedded in optimal cutting temperature medium. Sections, 5 μm thick, were prepared using a cryostat (CM 1900; Leica, Germany) collected at −20 °C and stored on positively charged glass slides (Thermo Scientific, Waltham, MA, USA). Serial sections, were rehydrated and subjected to immunofluorescence analysis. Nonspecific binding sites were blocked with 1% bovine serum albumin in phosphate-buffered saline (PBS) for 30 min at room temperature. Sections were then incubated with the primary antibodies AQP2 and Gαi3 overnight at 4 °C in saturation buffer. After washing in PBS, sections were incubated with the appropriate AlexaFluor-conjugated secondary antibodies (Life Technologies) for 30 min at room temperature washed and mounted onto glass slides with Mowiol. 

Confocal images were obtained with a confocal microscope (TSC-SP2, Leica; Wetzlar, Germany).

### 2.7. Water Permeability Video Imaging Measurements

Osmotic water permeability was measured by Video Imaging experiments. MCD4 cells were grown on 40 mm glass coverslips and loaded with 10 µM membrane permeable Calcein green-AM for 45 min at 37 °C, 5% CO_2_ in DMEM. Cells were incubated in the absence or presence of PTX (2 µg/mL) for 3 h at 37 °C and either stimulated with Forskolin (100 µM) for 30 min or left under basal conditions.

Alternatively, cells were grown as described before and were left under basal condition or stimulated with 100 µM Forskolin for 30 min, 10 µM quinidine for 45 min or 100 µM Forskolin and 10 µM quinidine.

The coverslips with dye-loaded cells were mounted in a perfusion chamber (FCS2 Closed Chamber System, BIOPTECHS, Butler, PA, USA) and measurements were performed using an inverted microscope (Nikon Eclipse TE2000-S microscope) equipped for single cell fluorescence measurements and imaging analysis. The sample was illuminated through a 40× oil immersion objective (numerical aperture NA = 1.30). The Calcein Green-AM loaded sample was excited at 490 nm. Emitted fluorescence was passed through a dichroic mirror, filtered at 515 nm (Omega Optical, Brattleboro, VT, USA) and captured by a cooled ECCD camera (CoolSNAP HQ, Photometrics, Tucson, AZ, USA). Fluorescence measurements, following iso- (290 mOsm; 140 mM NaCl, 5 mM KCl, 1 mM MgCl_2_, 1 mM CaCl_2_, 10 mM Hepes, 5 mM Glucose) or hyperosmotic (460 mOsm; isoosmotic solution added with 135 mM Mannitol) solutions, were carried out using Metafluor software v7.8.1.0 (Molecular Devices, MDS Analytical Technologies, Toronto, ON, Canada). The time course of cell shrinkage was measured as time constant (*Ki*, s^−1^), a parameter directly correlated to membrane water permeability. Statistical Analysis Data are reported as mean values ± S.E.Ms.

Statistical analysis was performed by one-way ANOVA (Analysis of Variance) followed by Newman-Keuls Multiple Comparison test with P.

### 2.8. Fluorescence Vesicles Swelling Assay

A fluorescence based assay of vesicles volume changes was carried out using a benchtop fluorescence plate reader with integrated liquid handling (FlexStation 3, Molecular Devices, MDS Analytical Technologies, San Jose, CA, USA) equipped to analyse real time fluorescence kinetic data in the 96-well format. The instrument consists of an incubated cabinet with fluorometer and integrated 96 channel pipettor which can transfer compounds from one microplate to the assay plate, allowing rapid kinetic assays.

A 96-well black walled microplate (Corning-Costar Corp., Corning, NY, USA) was pre-treated with Cultrex Basement Membrane Extract (BME) at 37 °C for 1 h to form a reconstituted basement membrane, then the excess was removed and vesicles (1 µg/µL) were seeded in the wells and incubated at 37 °C for 1 h. After that, the wells were washed with PBS to remove the unattached vesicles, replaced with K-MES buffer (100 mM Mes, 25 mM KCl, pH 6.5) and incubated with 67 µM R18 (Octadecyl Rhodamine B chloride, Biotium, Fremont, CA, USA) for 5 min at 37 °C. After rinsing in K-MES buffer, the 96-well plate was transferred into the plate-reader for the fluorescence assay. Fluorescence signal from vesicles, labelled with R18 at self-quenching concentration, was excited at 560 nm and detected at 590 nm. Fluorescence in each well was recorded continuously for 25 s (baseline), then for 50 s after rapid automated addition of 20 µM of Mas7 (diluted in K-MES). R18 fluorescence signal is directly related to vesicles volume changes.

Data acquisition was performed using SoftMax Pro software v5.3 (Molecular Devices, MDS Analytical Technologies, San Jose, CA, USA) and the data were analysed with Prism software (GraphPad software, San Diego, CA, USA). The time constant of vesicle volume variation was obtained by fitting the data to an exponential function.

### 2.9. Electron Microscopy

For electron microscopy, re-suspended vesicle fractions were fixed in 2.5% glutaraldehyde in 0.1 M in PBS, at pH 7.4, for 2 h at 4 °C. A drop of 5 μL of vesicle fractions were put on clean Parafilm and the grids (Formvar-carbon coated, 200 mesh Ni), were floated on the drop with their coated side facing the suspension for 10 min. The grids were transferred first to the drops of washing buffer PBS for 3 min and subsequently on the fresh drops of distillated water and washed for 4 min. For contrast enhancement, the grids were put on the 50 μL drop of 2% uranyl acetate. After the excess fluid was blotted from the grids by filter paper they were observed under an electron microscope.

### 2.10. Atomic Force Microscopy

AFM was used to obtain images of the vesicles topography. In an AFM a very sharp tip, attached to a flexible lever, interacts with the sample surface providing information on its morphology. Data were acquired using a Multimode VIII equipped with a Nanoscope V controller (Bruker) with the probe and the sample immersed in liquid. The AFM was operated in tapping mode with a lever oscillating at a frequency of 7 kHz (*k* = 0.24 N/m, SNL-10 Bruker, Berlin, Germany) to avoid sample damage. Few drops of a solution containing isolated vesicles were placed on freshly cleaved mica. The mica disk was placed in a humid chamber for 15 min to allow the vesicles to deposit. Then, the sample was washed with K-MES buffer to remove the unattached vesicles and was placed on the AFM stage. Vesicles were left under basal condition using K-MES buffer (100 mM Mes, 25 mM KCl, pH 6,5), or pre-treated with quinidine (0.5 mM) or DIDS (10 µM) or HgCl_2_ (0.3 mM) for 3 min and the sample topography was imaged. These images were considered as control images, then Mas7 (20 µM diluted in K-MES) was added and the same area on the sample was imaged to evaluate variations in the topography. An ‘ad hoc‘ algorithm was elaborated to analyse the same area before and after the addition of a given drug and calculation of each vesicle diameter, height and volume.

The structural features of each individual vesicle (centroid, isocontours elliptically shaped, volume, diameter, amplitude) were obtained by least-squares fitting of the AFM data using a two-dimensional elliptical Gaussian function:

(1) $$F\left(x,y\right)=Z_{0}+\mathrm{Aexp}\left\{\left(\frac{-1}{2\left(1-{\mathrm{cor}}^{2}\right)}\right)\left[{\left(\frac{x-x_{0}}{\sigma_{x}}\right)}^{2}+{\left(\frac{y-y_{0}}{\sigma_{y}}\right)}^{2}-\left(\frac{2\mathrm{cor}\left(x-x_{0}\right)\left(y-y_{0}\right)}{\sigma_{x}\sigma_{y}}\right)\right]\right\}$$

where Z_0_ is the baseline, A is the height of the peak, (x_0_, y_0_) is the centroid position of the vesicle, (α_x_, α_y_) represent the standard deviation of the peak and cor is the cross-correlation term respectively. The cor parameter ranges between −1 to 1 and is related to the asymmetry of the vesicle: a centro-symmetric vesicle has a cor value equal to zero. The vesicle profile estimation, by a two-dimensional elliptical Gaussian non-linear fit function, allows volume calculation of each vesicle using the following expression:

(2) $$\mathrm{Volume}=2\pi A\sigma_{x}\sigma_{y}\sqrt{1-{\mathrm{cor}}^{2}}$$

### 2.11. Membrane Fusion Assay

Membrane fusion assay was performed as described [32]. Briefly, vesicles (120 μg) were resuspended in 600 μL of K-MES buffer (100 mM Mes, 25 mM KCl, pH 6.5) and incubated with 2 µL of a 20 mM stock solution of R18 (Octadecyl Rhodamine B chloride) for 5 min at 37 °C. The mixture was centrifuged at 900× *g* for 10 min and the obtained pellet was resuspended in 400 μL of K-MES buffer. Thus, fluorescence signal from vesicles, labelled with R18 at self-quenching concentration, was recorded using a fluorimeter (RF-5301PC, Shimadzu Corporation, Kyoto, Japan) at excitation and emission wavelengths of 560 and 590 nm, respectively.

After 2 min vesicles were left under basal condition or incubated with 0.3 mM HgCl_2_ or 0.5 mM quinidine for 3 min under continuous stirring. Later, unlabelled plasma membrane (20 μg) fractions were added and fluorescence was recorded for additional 2 min under continuous stirring. Addition of cytosol (200 μg) lead to an increase of fusion due to a relief of R18 self-quenching and an increase of fluorescence signal.

To test whether chemical K^+^ gradient is the driving force promoting vesicle swelling as a consequence of TASK-2 gating, a set of experiments have been performed in TMA-MES buffer (100 mM MES, 25 mM Tetramethylammonium chloride, pH 6.5) instead of K-MES thus with a solution having the same osmolality as K-MES in which K^+^ ions have been substituted with TMA. This substitution abrogates the inwards K^+^ gradient (from outside to the vesicle lumen).

### 2.12. Apical Surface Biotinylation

MCD4 cells were cultured on 6-well filters for 4 days and were left under basal condition or stimulated with 100 µM Forskolin for 30 min, 10 µM quinidine for 45 min or 100 µM Forskolin and 10 µM quinidine.

Cells were washed thoroughly with ice cold Coupling Buffer (0.1 M NaPO_3_, 0.15 M NaCl in PBS, pH 7.2) before being subjected to oxidation with Na-meta-periodate 20 mM in Coupling Buffer for 30 min on ice in the dark. Following three rounds of washing with Coupling Buffer, cell surface glycoproteins were labelled with 5 mM Biocytin Hydrazide (EZ-Link^®^ Pierce) for 30 min.

The biotinylation buffer was removed, cells were incubated with Quenching Solution (50 mM NH_4_Cl in PBS, pH 7.2) for 5 min and washed three times with Coupling Buffer. Cells were solubilized with lysis buffer (1% Triton-X 100, 0.01% SDS in PBS), in the presence of proteases (1 mM PMSF, 2 mg/mL leupeptin and 2 mg/mL pepstatin A) and phosphatases (10 mM NaF and 1 mM sodium orthovanadate) inhibitors. The lysates were homogenized using ultrasonic homogenizer at 40 Hz for 20 s and centrifuged at 12,000× *g* for 20 min. An aliquot of the supernatants (20 μL) was diluted in Laemmli buffer while the remaining biotinylated proteins were pulled down with immobilized streptavidin beads for 1 h under rotation at 4 °C. The complexes were washed 3 times with wash Buffer (0.5% Triton-X 100, 0.01% SDS in PBS), denatured in Laemmli buffer for 10 min at 95 °C and analysed by SDS-PAGE and immunoblotted.

### 2.13. ^1^H-NMR Spectroscopy

**Scheme 1:**
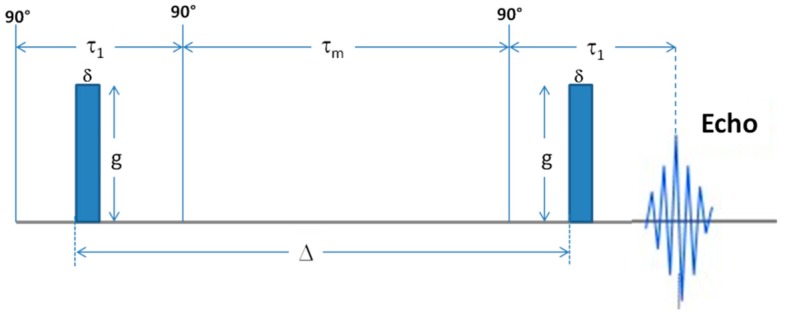
Schematic representation of the PFG-STE sequence.

NMR measurements were performed on a Bruker AVANCE 300 Wide Bore spectrometer (Berlin, Germany) working at 300 MHz on ^1^H. The employed probe was a Diff30 Z-diffusion 30 G/cm/A multinuclear with substitutable RF inserts, suitable to measure the self-diffusion coefficients by the Pulsed Field Gradient (PFG) method. Specifically, the PFG-stimulated-echo (PFG-STE) sequence (see Scheme 1) [33], used in this work, consists of three 90° rf pulses (π/2-τ_1_-π/2-τ_m_-π/2) and two gradient pulses (with magnitude g, duration δ and time delay Δ) that are applied after the first and the third rf pulses, respectively. The echo is found at time τ = 2τ_1_ + τ_m_.

The FT echo decays were analysed by means of the relevant Stejskal–Tanner expression:

(3) $$I\left(\tau \right)=I_{0}e^{-\beta D}$$

I and I_0_ represent the intensity/area of a selected resonance peak in the presence and in absence of gradients, respectively. β is the field gradient parameter, defined as $\beta ={\left(\gamma g\delta \right)}^{2}\left(\Delta -\frac{\delta }{3}\right)$; D is the measured self-diffusion coefficient. For the samples analyzed, the used experimental parameters were Δ = 20 ms, δ = 1 ms and the gradient amplitude, g, varied from 10 to 300 G cm^−1^ incremented in 10 steps. The number of scans was 8. Based on the repeatability of the measurements and on the very low standard deviation of the fitting curve, the uncertainty on the self-diffusion measurements is less than 0.5%.

^1^H spectra were obtained by applying the Fourier transform to the resulting free-induction decay (FID) of a single π/2 pulse sequence, with a pulse length of 10 µs. The spin-lattice relaxation time (T_1_) was also measured by the inversion recovery sequence to set the delay time after each acquisition scan. T_1_ was find to be quite long, that is, 2.3 s, however, to quickly conduct diffusion measurements, a delay time of 3 s was used. Spectra and diffusion measurements were performed at RT on the vesicles sample before the addition of the Mas7 and soon after. Spectra were referenced against pure water set at 0 ppm and were acquired with the same number of scans (8 scans), in order to compare their intensities. The process was followed for more than one hour.

Isolated vesicle suspension was prepared and allowed to sediment in ice, the supernatant removed to achieve a very dense vesicle suspension for NMR analysis. About 200 µL of vesicles suspension was inserted into the 5 mm NMR tube using a syringe; ^1^H spectra and diffusion measurements were acquired (data at time *t* = 0); next 25 µL of Mas7 (20 μM) was added in the tube and gently mixed with a syringe. A proton spectrum was acquired and a D measurement was recorded very quickly (data at time *t* = 1 min). D measurements and spectra acquisitions were executed in succession for circa 60 min.

### 2.14. Measurement of Intravesicular K^+^ Concentration

To measure intravesicular K^+^ concentration, 400 μL of vesicle suspension (containing 940 μg of proteins) were spun at 200,000× *g* in a Beckman Rotor TLA 120.1 for 1 h at 4 °C. Pelletted vesicles were resuspended in distilled water and sonicated at 60 Hz for 20 s, to disrupt vesicular structure. The suspension was spun at 200,000× g for 1h at 4 °C, to remove the membrane vesicles and the obtained supernatant (at final volume of 1 mL with distilled water), was used to measure the intravesicular K^+^ concentration.

The determination of K^+^ in the samples was carried out utilizing Inductively Coupled Plasma Mass Spectrometry (Elan DRC-e ICP-MS instrument Perkin-Elmer SCIEX, Norwalk, CT, USA). The sample delivery system was as previously described [34]. Quantitative analysis was performed processing the calibration curve of K^+^. Briefly, 250 µL of sample was transferred into a graduate polypropylene test tube, diluted to 50 mL with ultrapure water and stabilized with HNO_3_ 65%. Blank was prepared in the same way. The calibration standards were prepared by dilution of a Multielement Calibration Standard 3 containing Potassium at 10 mg/L. The calibration curve of potassium was obtained processing six points at different concentration in the calibration range of 10–2000 µg/L. The HNO_3_ 65% used for the preparation of samples and calibration standards was Suprapur (Merck, Darmstadt, Germany). The water, with a resistivity of 18.2 MΩcm, was obtained from a Milli-Q plus system (Millipore, Bedford, MA, USA). The multielement calibration standard used was PerkinElmer Pure Plus (USA).

The number of vesicles present in the sample was calculated according to the estimation that that the weight of a vesicle is composed of 1/3 protein, 1/3 lipid, water and so forth [35]. Since 1 gr of vesicles contains about 10^17^ vesicles, 940 μg of vesicles contain 9.40 × 10^13^ vesicles. Based on AFM study, the estimated volume of 1 vesicle is 1.13 × 10^−16^ mL, then 9.40 × 10^13^ vesicles have a total volume of 0.0106 × 10^−3^ L. Correlating this value with the measured K^+^ μM obtained by Inductively Coupled Plasma Mass Spectrometry, an estimated intravesicular K^+^ concentration of 5.3 mM is obtained. 

### 2.15. Statistical Analysis

All values are reported as means ± S.E.Ms. Statistical analysis was performed by one-way ANOVA followed by Newman-Keuls Multiple Comparison test with * *p* < 0.05 were considered statistically different. When applicable, paired t-test student was also used.

## 3. Results

### 3.1. Heterotrimeric G Protein Gαi3 Is Associated with AQP2 Vesicles in MCD4 Mouse Collecting Duct Cells

**Figure 1:**
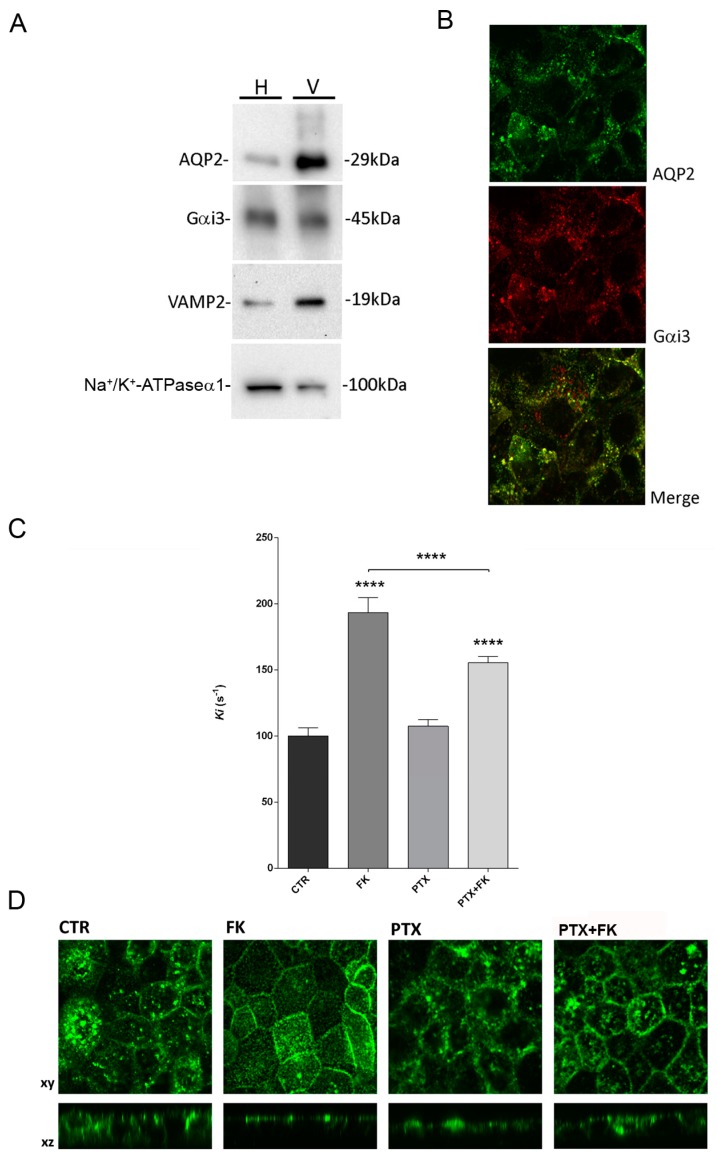
Inhibition of the Gαi3 protein, localized in AQP2 vesicles, prevents the Forskolin-induced increase in water permeability. (**A**) Expression of AQP2, Gαi3, Vamp2 and Na^+^/K^+^-ATPase in MCD4 cell homogenate and in AQP2 vesicles purified from MCD4. 30 μg of protein from total homogenate (H) or AQP2 vesicles (V) were blotted and probed with indicated antibodies. AQP2 is found highly enriched in the vesicle fraction along with the vesicle marker VAMP2, with low contamination by plasma membranes tested using the marker Na^+^/K^+^-ATPaseα1. Gαi3 appears to be expressed both in total homogenate and in vesicles fraction. Western blot is representative of 3 experiments. (**B**) Immunofluorescence localization of AQP2 and Gαi3 in polarized MCD4 cells. Representative confocal images showing substantial colocalization between AQP2 (green) and Gαi3 (red) evident upon overlay of individual fluorescence channels. (**C**) Effect of PTX treatment on osmotic water permeability under FK action in MCD4 cells. Cell monolayers were treated with Forskolin (100 μM) and/or PTX (2 μg/mL) as described in Experimental Procedures. The time course of fluorescence changes in calcein-loaded cells indicates that in cells treated with FK (100 µM) a significant increase in osmotic water permeability reported as *Ki* (193.3 ± 11.22 s^−1^, **** *p* < 0.0001, *n* = 58 cells) was observed, compared to control cells (100 ± 6.05 s^−1^, *n* = 35 cells). In contrast, when cells were treated with PTX the response to FK was significantly reduced (155.4 ± 4.54 s^−1^, **** *p* < 0.0001, *n* = 192 cells) with respect to FK action alone (values are reported as means ± S.E.Ms). (**D**) Effect of PTX on FK-induced AQP2 trafficking. Analysis by confocal microscopy revealed that under resting conditions AQP2 was mainly located intracellularly; stimulation of cells with FK resulted in an increased staining to the apical membrane. When cells were treated with PTX, the FK action was partially prevented.

We have previously shown that in rabbit renal collecting duct CD8 cells, a heterotrimeric G protein Gαi3 expressed in AQP2 bearing vesicles is required for the vasopressin-induced insertion of AQP2 in the apical membrane [28]. Here, the expression of Gαi3 was evaluated in mouse renal collecting duct hAQP2 expressing MCD4 cells [31]. Purified AQP2 vesicles isolated from MCD4 cells were first biochemically characterized by immunoblotting using specific markers. With respect to total MCD4 homogenate, AQP2 was found enriched in isolated vesicles along with VAMP2, a specific marker of AQP2 vesicles [36]. In contrast, Na^+^/K^+^-ATPase, a marker of the plasma membrane, had a very low expression in this fraction (Figure 1A). The heterotrimeric G protein Gαi3 was found expressed both in the total homogenate and in AQP2 vesicles thus reflecting a broad expression in several cellular compartments as previously reported in renal cells [28] (Figure 1A).

The subcellular distribution of Gαi3 protein in MCD4 cells was then evaluated by immunolocalization and analysed by confocal microscopy. Gαi3 appeared mainly localized intracellularly displaying a significant level of co-localization with AQP2 (Figure 1B).

### 3.2. Gi inhibition with PTX Prevented the Forskolin-Induced Increase in Water Permeability

The functional involvement of Gαi3 in AQP2 trafficking was next evaluated using the pertussis toxin (PTX 2 µg/mL for 3 h at 37 °C), which causes ADP-ribosylation of the Gi protein thus uncoupling Gi proteins from their cognate receptors. Cell monolayers were loaded with membrane permeable Calcein-AM. Fluorescence measurements were carried out in the presence of hyperosmotic solutions. Forskolin (FK 100 µM, for 30 min at 37 °C) treatment induced a significantly higher temporal osmotic response reported as *Ki* (193.3 ± 11.22 s^−1^, *n* = 58, **** *p* < 0.0001) as compared to untreated cells (100 ± 6.05 s^−1^, *n* = 35). In contrast, in cells pre-treated with PTX the response to FK was significantly reduced (155.4 ± 4.54 s^−1^, *n* = 192, **** *p* < 0.0001). PTX alone had no effect on the basal osmotic water permeability (Figure 1C). 

Immunolocalization and subsequent analysis by confocal microscopy confirmed that in cells pre-treated with PTX, AQP2 relocation to the apical plasma membrane was partially prevented (Figure 1D). These data suggest that a Gi protein sensitive to PTX is required for AQP2 trafficking.

### 3.3. The Heterotrimeric G protein Gαi3 Is Associated with AQP2 Vesicles in Rat Kidney Inner Medulla

To evaluate the actual functional role of Gαi3 in AQP2 trafficking, AQP2 bearing vesicles were isolated from rat inner medulla representing a native tissue therefore overcoming possible artefacts due to the overexpression of AQP2 in intracellular compartments in MCD4 cells. Isolated vesicles were biochemically characterized for the expression of marker proteins. Isolated AQP2 vesicles (V) were blotted, probed with different antibodies and the immunodetected signal intensity compared to total homogenate (H). 

**Figure 2:**
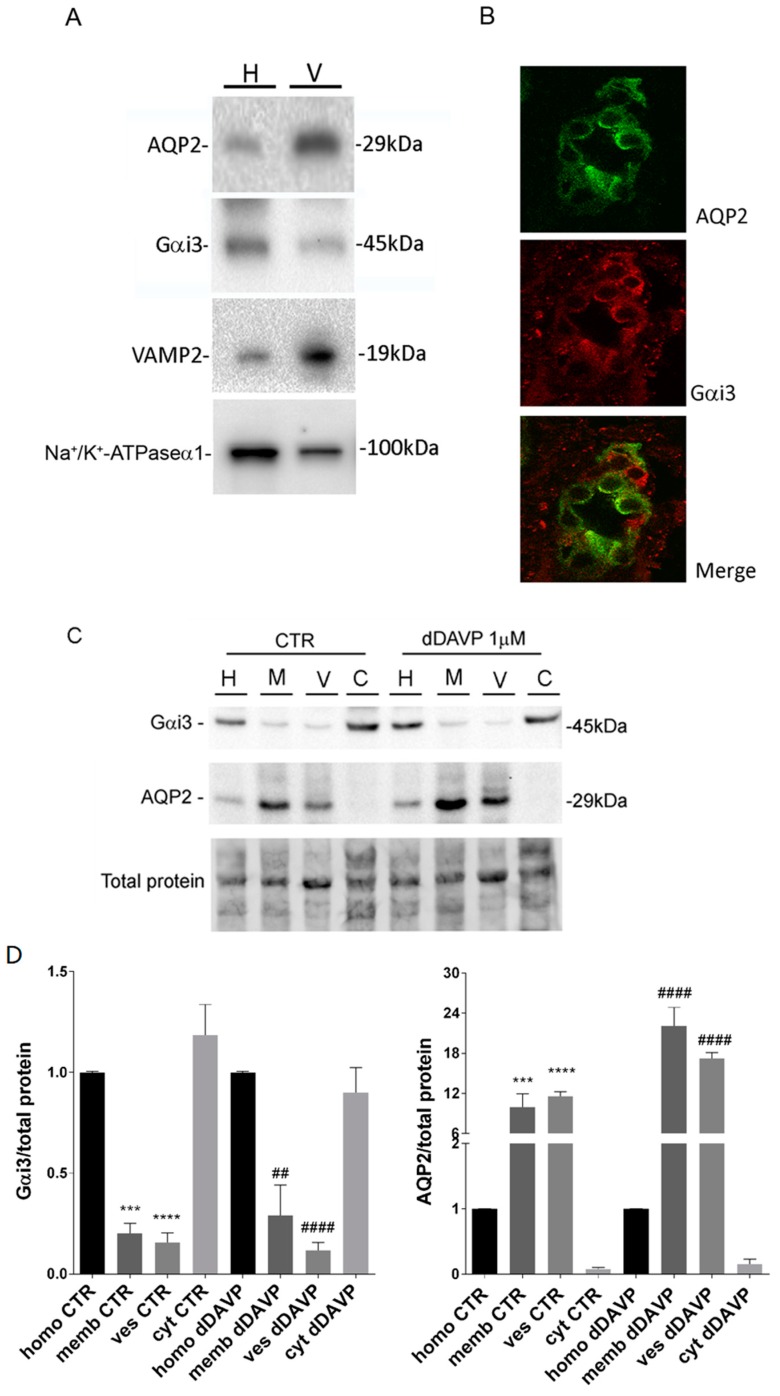
Heterotrimeric G protein Gαi3 is associated with AQP2 vesicles isolated from rat kidney medulla. (**A**) Expression of AQP2, Gαi3, Vamp2 and Na^+^/K^+^-ATPase in rat kidney medulla homogenate and AQP2 vesicles isolated from rat kidney medulla. 30 μg of protein from total rat homogenate (H) or purified AQP2 vesicles from rat inner kidney medulla (V) were blotted and probed with indicated antibodies. AQP2 is found highly enriched in the vesicle fraction along with the vesicle marker VAMP2, with low contamination by plasma membranes tested using the marker Na^+^/K^+^-ATPaseα1. Gαi3 appears to be expressed both in total homogenate and in vesicles fraction. Western blot is representative of 3 experiments. (**B**) Localization of AQP2 and Gαi3 in rat kidney. Representative confocal images showing substantial colocalization between AQP2 (green) and Gαi3 (red) evident upon overlay of individual fluorescence channels. The two proteins display a predominant punctuate intracellular localization. (**C**) Subcellular distribution of Gαi3 and AQP2 under basal condition or stimulated with dDAVP (1µM for 45 min) in rat kidney. Rat kidney slices were stimulated with dDAVP or left under basal conditions, homogenized and subjected to differential centrifugation to obtain plasma membrane, intracellular vesicles and cytosol. Protein samples were blotted and probed with specific antibodies against AQP2 and Gαi3. (**D**) Statistical analysis of Gαi3 and AQP2 expression normalized to total protein in membrane (M), vesicle fraction (V) and total homogenate (H) is reported. Values are reported as means ± S.E.Ms; *** *p* < 0.001; **** *p* < 0.0001 versus homogenate CTR; *n* = 3; ^##^ *p* < 0.01; ^####^ *p* < 0.0001 versus homogenate dDAVP; *n* = 3.

With respect to total MCD4 homogenate, AQP2 was found co-enriched in isolated vesicles along with VAMP2. Conversely, Na^+^/K^+^-ATPase, a marker of the plasma membrane, had a low expression in this fraction (Figure 2A). As for MCD4 cells, the heterotrimeric G protein Gαi3 was found expressed both in the total homogenate and in AQP2 vesicles consistent with a broad expression in several cellular compartments.

Immunofluorescence studies revealed that AQP2 and Gαi3 in the isolated rat kidney medulla display a substantial co-expression in many cells. The staining of both proteins appeared mainly located intracellularly (Figure 2B). 

Since, by western blotting Gαi3 appear predominantly expressed in the homogenate, cell fractionation experiments were performed to assess the subcellular distribution of Gαi3 and AQP2 before and after vasopressin (dDAVP) stimulation. Obtained results revealed that Gαi3 has a major expression in the cytosol and dDAVP stimulation causes a very slight redistribution of Gαi3 which remained mainly expressed in the cytosol with an small enrichment in the plasma membrane fraction. Conversely, as expected, AQP2 is mainly expressed in intracellular vesicles and plasma membrane and vasopressin stimulation results in a strong redistribution of AQP2 to the plasma membrane fraction (Figure 2C).

### 3.4. Gi Activation with Mastoparan (Mas7) Increases AQP2 Vesicle Size

#### 3.4.1. Fluorescence-Based Assay for AQP2 Vesicle Swelling

**Figure 3:**
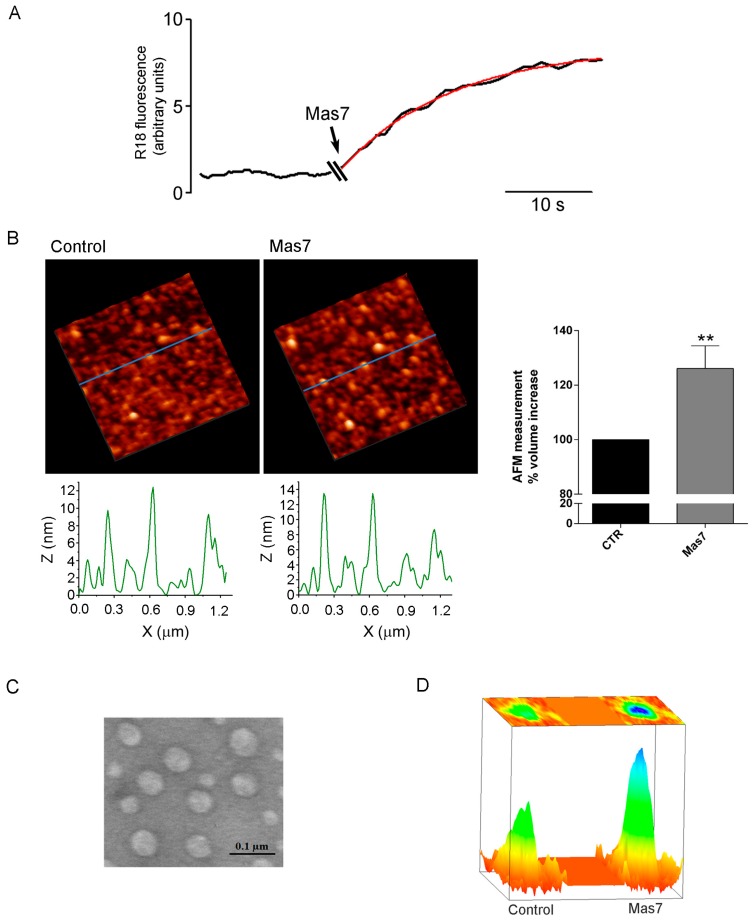
Activation of Gi protein, located in AQP2 vesicles, promotes vesicles swelling. (**A**) Time course of AQP2 vesicles swelling assay monitored using the fluorescent lipid probe R18 loaded at self-quenching concentration. Vesicles plated on multiwells were labelled with R18 and exposed to Mas7 (arrow) in the absence of an osmotic gradient. The swelling process was depicted by an increase in the fluorescent emission signal with a maximal signal achieved within the first 30 s. The exponential fit is shown in red. No swelling was observed in the absence of Mas7 (**B**) 3D AFM images and section analysis of isolated AQP2 vesicles under basal conditions and after exposure to Mas7. Size changes of each single vesicle were monitored and processed using an algorithm specifically elaborated for this purpose (see Experimental Procedures for details). Statistical analysis of vesicle size changes, on the right, shows that exposure of AQP2 vesicles to Mas7 in the absence of an osmotic gradient resulted in a significant volume increase with respect to resting condition. Data (means ± S.E.Ms) were analysed by Student’s t test. ** *p* = 0.0065. (**C**) Electron microscopy of isolated vesicles showing a pretty homogeneous vesicle size of 60 nm diameter in average. (**D**) Representative example of reconstructed 3D AFM image of the same vesicle before and after exposure to Mas7 depicting the volume increase with color-coded height information in a three-dimensional perspective. On top is reported the projection on the x-y plane of the three-dimensional AFM vesicle image. This 3D Colormap surface with projection, was obtained by plotting the data twice: once as a 3D surface plot and a second time as a flat surface.

To monitor vesicles swelling in real time, a fluorescent based assay was used allowing to record the fluorescence signal from vesicles plated on multiwells. Vesicles were labelled with R18 (Octadecyl Rhodamine B chloride), a fluorescent lipid probe, at self-quenching concentration. As a consequence of swelling, R18 dilution is expected to occur, leading to an increase in the fluorescent emission signal. With this technique the rate of vesicles volume changes can be monitored, in response to a specific treatment. AQP2 isolated vesicles were treated with Mas7, an active mastoparan analogue [37], known to activate Gi protein. The results showed that Mas7 induced a significant increase in the fluorescent emission signal quantified in terms of the time constant 1/τ (0.127 ± 0.018 s^−1^; *n* = 18) compared to untreated vesicles, suggesting that AQP2 vesicle swelling is promoted by the activation of an endogenous Gi protein (Figure 3A). The time course of vesicles volume change indicated that the swelling reached its maximum amplitude within 30 s.

#### 3.4.2. Single AQP2 Vesicle Swelling Measured by Atomic Force Microscopy

For a precise evaluation of vesicle volume changes during treatments, several three dimensional images of vesicles at nanometre resolution were obtained by AFM. Vesicle suspension (in K-MES) was plated on mica and allowed to adhere for about 15 min. Vesicles were then imaged under this experimental condition (Figure 3B, control). Subsequently the bathing solution was replaced with a solution containing 20 μM Mas7 in K-MES (Figure 3C, Mas7). The same area of vesicles plated on mica was imaged before and after Mas7 treatment using an ‘ad hoc’ algorithm developed to evaluate changes in a single vesicles size. Mas7 treatment was associated with a significant increase in vesicle volume estimated as 34% (** *p* = 0.0065, *n* = 16) (Figure 3B). Specifically, for the calculation of single vesicle volume, the height and the diameter of each single vesicle was measured before and after treatment with Mas7. Mas7 caused an increase in diameter (from 58.0 ± 5.9 nm to 67.1 ± 7.3 nm, *n* = 16) and height (from 23.7 ± 2.4 nm to 25.4 ± 2.1 nm, *n* = 16). Analysis by electron microscopy confirmed that isolated vesicles have an average diameter of 60 nm (Figure 3C). A representative example of reconstructed 3D AFM image of the same vesicle before and after exposure to Mas7 is shown in Figure 2D depicting the volume increase of a single vesicle with color-coded height information in a three-dimensional perspective. On top is reported the projection on the x-y plane of the three-dimensional AFM vesicle image (Figure 3D).

Through a direct imaging of vesicles at nanoscale level, these data confirm that activation of a vesicles associated-Gi with Mas7 causes a significant increase in vesicle size.

*NMR-Diffusometry*. Vesicle swelling implies variation of the water diffusion into the vesicle. Water self-diffusion coefficient (D) is one of the most important and fundamental physico-chemical quantity which provides information on the dynamics and structure of nano-assemblies in aqueous solutions. This can be achieved by measuring the molecule’s mean square displacement <z^2^> in a time Δ, by the PFG-NMR method (spin-echo or stimulated-echo sequences), a technique also known as NMR-Diffusometry [38]. This non-invasive and convenient technique was therefore applied to investigate water diffusion and the stability of the phenomenon in vesicles after exposure to Mas7. Specifically, the variation of D in the vesicles solution reflects the swelling process, while the information on stability is obtained by monitoring how this coefficient changes with time.

**Figure 4:**
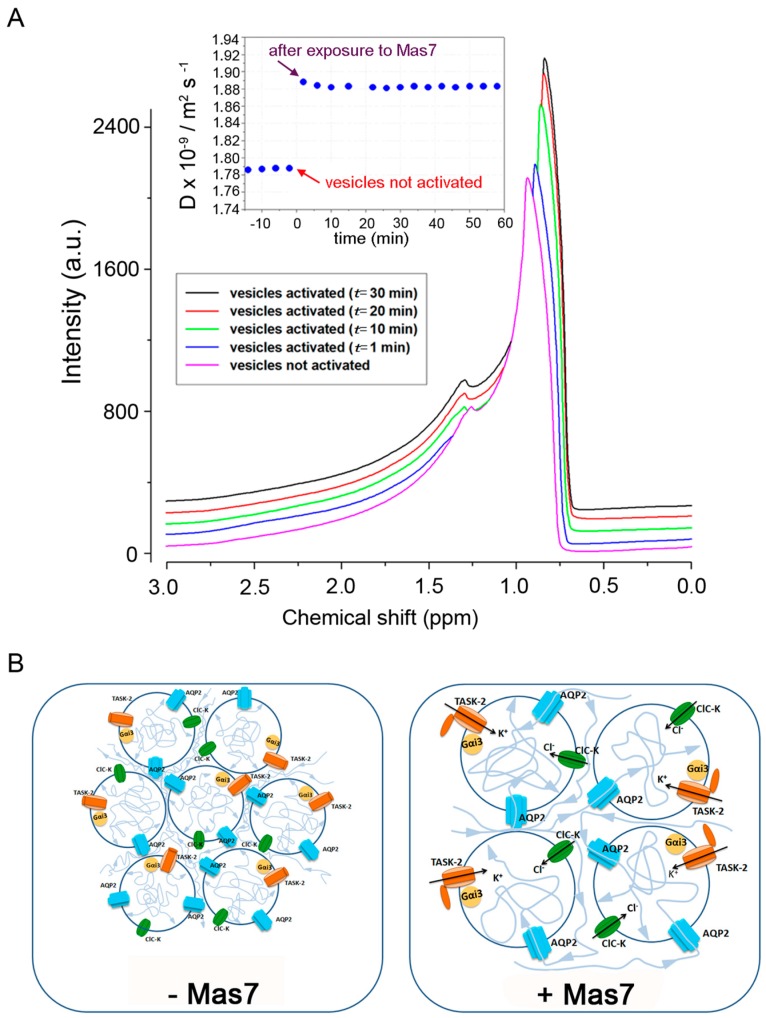
NMR-Diffusometry. Water self-diffusion coefficient D measurement before and after addition of Mas7 (A inset). (**A**) Before activation with Mas7, the measured ^1^H diffusion coefficient D of the vesicles solution was (1.78 ± 0.01) × 10^−9^ m^2^ s^−1^. After the addition of Mas7, D value monitored at the time *t* = 1 min was equal to (1.89 ± 0.01) × 10^−9^ m^2^ s^−1^ and reached a steady state at a constant value for the next 60 min. ^1^H spectra analysis shows the time evolution of the proton spectrum before and soon after the activation process of the vesicles. With respect to pure water the chemical shift of the vesicles suspension signal is found at lower field, is quite broad (FWHM ~190 Hz) and very asymmetric, suggesting multiple water environments that are not in rapid (on the NMR time scale) equilibrium with each other. Confinements of water molecules are consistent with a complex vesicular system wherein water inside and between the vesicles is present. Under these conditions the greatest contribution to the signal derives from water between the vesicles, as it is expected to be much higher. Upon Mas7 addition, a shift on the right of the signal is observed (blue line, spectrum at *t* = 1 min) and this shifting continues for about 10 min after which it remains stable, reflecting the same time course of D changes. (**B**) Proposed model: after exposure to Mas7, vesicle swelling reduces the packing by creating more empty spaces, therefore the water mobility between the vesicles grows as consequence of less tortuousness of the diffusive path and lower obstruction factor.

Before activation with Mas7, the measured ^1^H diffusion coefficient (D) of the vesicles solution was (1.787 ± 0.002) × 10^−9^ m^2^ s^−1^ (error was estimates by semi-dispersion calculated on the set of performed measurements). After the addition of Mas7, D was monitored starting from the time *t* = 1 min (time elapsed during the operations of placing the sample in the tube and then in the NMR probe). The D value measured at this time-point was equal to (1.885 ± 0.003) × 10^−9^ m^2^ s^−1^ with an estimated 6% increase in the D value and reached a steady state at a constant value for the next 60 min (Figure 4A, inset). Vesicle preincubation with HgCl_2_ (a known water channel inhibitor) before Mas7 treatment, resulted in a significant reduction in D (from (1.722 ± 0.001) to (1.775 ± 0.005) × 10^−9^ m^2^ s^−1^) corresponding to about 3% decrease in the D value compared to that measured under control conditions suggesting a contribution of AQP-mediated water permeability in vesicle swelling.

Structural changes of the vesicles secondary to exposure to Mas7 were also supported by the ^1^H spectra analysis, shown in Figure 4A, wherein the time evolution of the proton spectrum before and soon after the activation process of the vesicles is detected. With respect to pure water (set at 0 ppm), the chemical shift of the vesicles suspension signal is found at lower field, is quite broad (Full Width at Half Maximum, FWHM ~190 Hz) and very asymmetric, suggesting multiple water environments that are not in rapid (on the NMR time scale) equilibrium with each other. Confinements of water molecules are consistent with a complex vesicular system wherein water inside and between the vesicles is present. Under these conditions the greatest contribution to the signal derives from water between the vesicles, as it is expected to be much higher (Figure 4A and proposed model in B).

Upon Mas7 addition, a shift on the right of the signal is observed (blue line, spectrum at *t* = 1 min) and this shifting continues for about 10 min after which it remains stable, reflecting the same time course of D changes.

According to the above, the increase in water self-diffusion coefficient upon Mas7 addition can be explained taking into account the dimensional polydispersity of the vesicles which favours a certain packing (the dispersion used for this experiment is very dense and viscous); after exposure to Mas7, vesicle swelling reduces the packing by creating more empty spaces, therefore the water mobility between the vesicles grows as consequence of less tortuousness of the diffusive path and lower obstruction factor (see proposed model in Figure 4B)

Incubation with HgCl_2_, a known water channel inhibitor, partially prevented this increase consistent with inhibition of water movement through aquaporins (not shown). 

### 3.5. AQP2 Vesicles Express Potassium TASK-2 and ClC-K Channels

Obtained data using three different biophysical techniques to profile vesicle swelling, indicated that activation of a Gi expressed in the vesicles is associated with water entry into the vesicles, likely secondary to an inwardly osmotic gradient generated by ion diffusion into the vesicle lumen. Since Gαi3 has been shown to regulate chloride and potassium channels [6,39,40,41,42] we evaluated whether these channels were immunodetected in AQP2 isolated vesicles. 

Previous proteomic analysis of immunoisolated AQP2 vesicles identified some ion channels that are associated to vesicles. Among them, potassium channels subfamily K have been detected by LC-MS/MS-based proteomic analysis [43]. Specifically, peptide sequences were used to search in BLAST corresponding proteins and we identified TASK-2 as the potassium channel associated with AQP2 vesicles. TASK-2 is a member of the superfamily of potassium channel proteins containing two pore-forming domains. It is highly expressed in the kidney and has a strong dependence external pH [44]. TASK-2 has been proposed as the swelling-activated K channel responsible for the cell volume regulation process during osmolyte absorptions in the proximal tubules [45]. TASK-2 channel is regulated by G protein [46] making this channel a good candidate mediating vesicle swelling.

**Figure 5:**
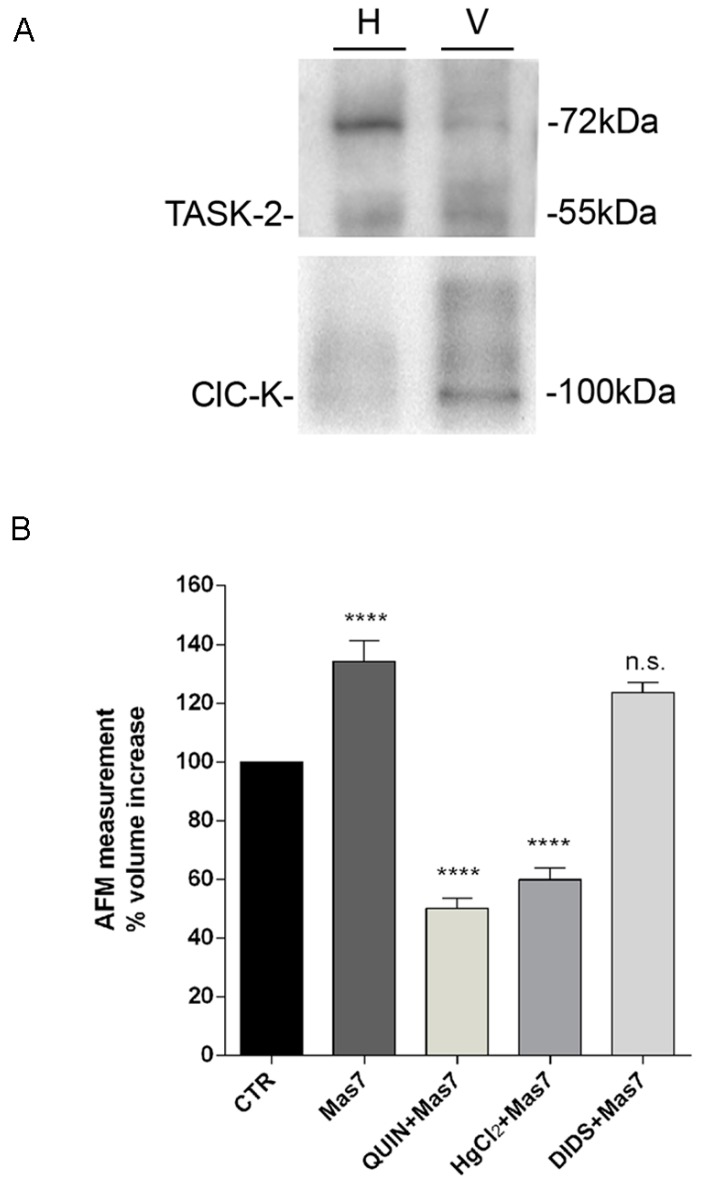
Molecular mechanism of AQP2 vesicle swelling. (**A**) Expression of TASK-2 and ClC-K in AQP2 vesicles. 30 μg purified AQP2 vesicles from rat inner kidney medulla were blotted and probed with specific anti-TASK-2 and with anti-ClC-K antibodies. Anti-TASK-2 antibodies detected a 55 kDa band along with a 72 kDa bands corresponding to the monomeric and glycosylated forms of the TASK-2 potassium channel respectively whereas anti-ClC-K antibodies stained a band at 100 kDa. (**B**) AQP2 vesicle swelling measured by Atomic Force Microscopy: effect of TASK-2 inhibitor. Means ± S.E.Ms values of % of volume changes measured by AFM with respect to control. Activation of Gi with Mas7 (20 µM) resulted in a 34% increase in vesicle volume (134.1 ± 7.1%, *n* = 16, **** *p* < 0.0001). Preincubation of vesicle with quinidine (0.5 mM) completely abolished Mas7-induced vesicle swelling causing instead a significant reduction in vesicle volume with respect to control (50.2 ± 3.4%, *n* = 63, **** *p* < 0.0001). Vesicle preincubation with HgCl_2_ (0.3 mM) also prevented Mas7 activated vesicle swelling reducing vesicle volume (59.9 ± 3.9%, *n* = 45, **** *p* < 0.0001). DIDS (10 µM) had no effect on Mas7 induced increase in vesicle volume (123.7 ± 3.3%, *n* = 100).

By immunoblot, specific anti-TASK-2 antibodies detected a 55 KDa band along with a 72 KDa bands corresponding to the monomeric and glycosylated forms of the TASK-2 potassium channel respectively [47,48]. The immunodected bands were found both in cell homogenate and in isolated AQP2 vesicles with an apparent higher expression in the homogenate (Figure 5A). Conversely, the chloride channel ClC-K (kidney-specific chloride channel) was immunodetected at 100 KDa both in the homogenate and in vesicles with a higher expression in vesicles (Figure 5A). 

### 3.6. Molecular Mechanism of AQP2 Vesicle Swelling

To test the hypothesis that vesicle swelling is due to Gαi3-dependent activation (by Mas7) of the K channel TASK-2 and/or Cl channel associated to vesicles, the effects of both channel inhibitors as well as of HgCl_2_, were tested at single vesicle level by AFM. Figure 5B shows the statistical analysis of AFM measurements of vesicle swelling expressed as percentage of control. Activation of Gi with Mas7 (20 µM) showed a 34% increase in vesicle volume (134.1 ± 7.1%, *n* = 16, **** *p* < 0.0001). Preincubation of vesicle with quinidine (0.5 mM) completely abolished Mas7-induced vesicle swelling causing instead a significant reduction in vesicle volume with respect to control (50.2 ± 3.4%, *n* = 63, **** *p* < 0.0001). Vesicle preincubation with HgCl2 (0.3 mM) also prevented Mas7 activated vesicle swelling reducing vesicle volume (59.9 ± 3.9%, *n* = 45, **** *p* < 0.0001). 

For both quinidine and HgCl_2_ treated vesicles, the decrease in vesicles volume corresponded also to a decrease in the lateral dimension as well as in the height of the vesicles (diameter from 27.99 ± 3.33 nm to 15.82 ± 0.82 nm and height 39.73 ± 11.97 nm to 19.86 ± 1.89 nm for quinidine; diameter from 45.28 ± 2.62 nm to 36.16 ± 2.30 nm, height from 19.76 ± 1.53 nm to 16.06 ± 1.4 nm for HgCl_2_).

Conversely, DIDS (10 µM) had no effect on Mas7 induced increase in vesicle volume (123.7 ± 3.3%, *n* = 100), suggesting that chloride channels are not implicated in the swelling process. In line, no significant variation of either diameter and height was recorded (diameter from 35.10 ± 2.11 nm to 40.89 ± 2.53 nm and height from 22.57 ± 1.45 nm to 23.17 ± 1.46 nm for DIDS).

### 3.7. Driving Force Behind the Vesicle Volume Increase: Measurement of Intravesicular K^+^ Concentration

Together obtained results indicate that TASK-2 gating results in K^+^ entry into the vesicles implying the existence of a K^+^ gradient between the external solution and the intravesicular lumen. To test the existence of such gradient, which provides the driving force behind the vesicle volume increase, the intravesicular K^+^ concentration in AQP2 vesicles was measured by Inductively Coupled Plasma Mass Spectrometry (see Methods for details). Obtained results revealed that the intravesicular K^+^ concentration is 5.3 mM. Since in the K-MES buffer used in our experiments, the K^+^ concentration is 25 mM there is an inwardly K^+^ chemical gradient, provided that TASK-2 is opened by mastoparan activation.

### 3.8. Inhibition of K Channels Prevents Forskolin-Induced Increase in Water Permeability

**Figure 6:**
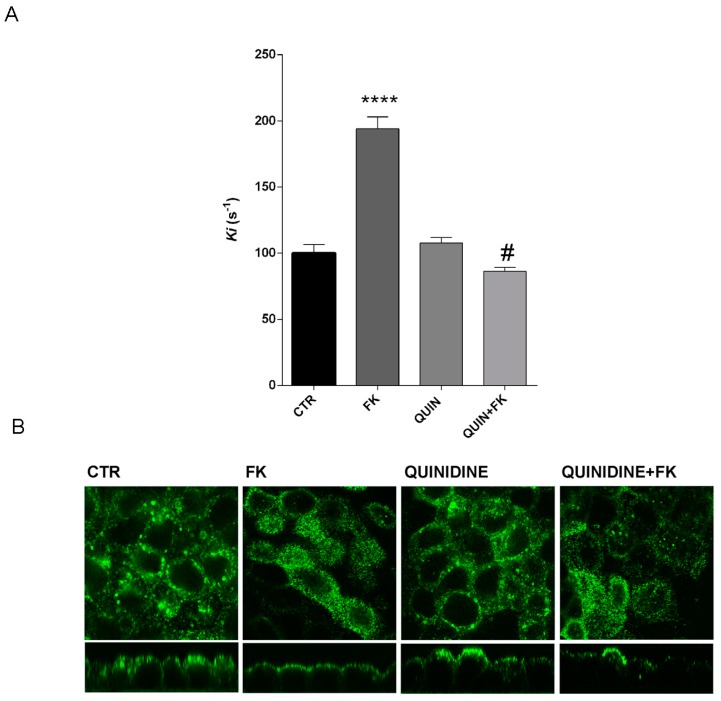
Effect of quinidine treatment on osmotic water permeability under FK action in MCD4 cells. (**A**) Cells were grown and treated with Forskolin (FK) 100 μM and/or quinidine (10 μM) as described in Experimental Procedures. Treatment with FK (100 µM) induced a significantly higher temporal osmotic response reported as *Ki* (values are reported as means ± S.E.Ms; 194 ± 9.04 s^−1^, **** *p* < 0.0001) as compared to untreated cells (100 ± 6.65 s^−1^). Quinidine (10 µM) caused a significant reduction of FK response (86.40 ± 2.83 s^−1^, # *p* < 0.0001). Quinidine alone had no effect on the basal osmotic water permeability. (**B**) Immunolocalization and analysis by confocal microscopy of AQP2 localization in polarized MCD4 cells. Under basal condition, AQP2 staining was mainly intracellular. FK stimulation caused AQP2 translocation to the apical plasma membrane. In quinidine treated cells AQP2 relocation to the apical plasma membrane in response to FK was partially prevented.

In renal collecting duct principal cells, AQP2 vesicles are targeted to the plasma membrane in response to vasopressin, via a cAMP/PKA mediated pathway, resulting in an increase in the osmotic water permeability. To test the functional involvement of TASK-2 in AQP2 trafficking in intact cells, cAMP-activated AQP2 trafficking was evaluated in cells treated with quinidine. First, the time course of osmotic response was tested in cells grown on glass coverslips loaded with membrane permeable Calcein-AM. Fluorescence measurements were carried out in the presence of hyperosmotic solutions. Treatment with the cAMP-elevating agent FK (100 µM) induced a significantly higher temporal osmotic response reported as *Ki* (194 ± 9.04 s^−1^, *n* = 118, **** *p* < 0.0001) as compared to untreated cells (100 ± 6.65 s^−1^, *n* = 106). In contrast, in cells pre-treated with quinidine (10 µM) the response to FK was significantly reduced (86.40 ± 2.83 s^−1^, *n* = 190, # *p* < 0.0001). Quinidine alone had no effect on the basal osmotic water permeability (Figure 6A). These data indicate that TASK-2 inhibition prevents the increase in AQP2-mediated water permeability in response to cAMP.

Immunolocalization and subsequent analysis by confocal microscopy revealed that under basal condition, AQP2 staining was mainly intracellular whereas FK stimulation caused AQP2 translocation from an intracellular pool to the apical plasma membrane. In quinidine treated cells, AQP2 relocation to the apical plasma membrane in response to FK was partially prevented (Figure 6B).

To verify whether the apical localization observed by confocal studies coincided with a real insertion of AQP2 into the membrane, cell surface biotinylation experiments were performed.

**Figure 7:**
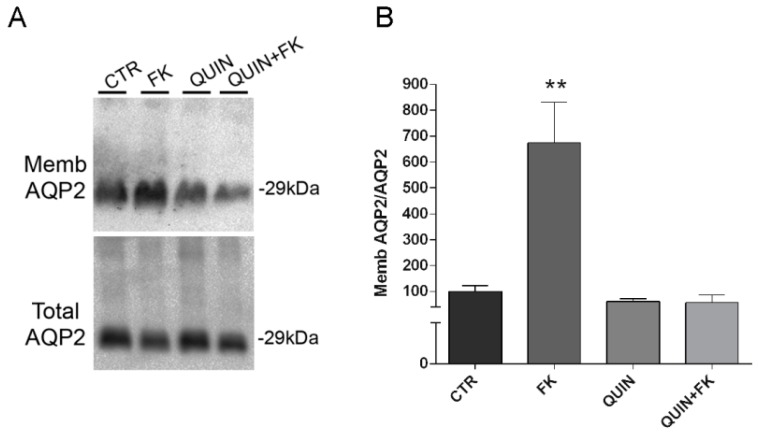
Cell surface biotinylation of AQP2 in MCD4 cells. (**A**) Immunoblotting analysis of total and apical AQP2 under different experimental conditions. Compared to untreated cells (CTR), FK significantly increased the abundance of AQP2 at the apical plasma membrane. Quinidine impaired FK-stimulated increase in cell surface expression of AQP2. Quinidine alone did not affect cell surface expression of AQP2. (**B**) Densitometric analysis of the 29 kDa biotinylated AQP2 band normalized to total AQP2 (values are reported as means S.E.Ms; ** *p* = 0.006 versus CTR).

To increase the yield of the detectable membrane-inserted AQP2, the cell surface was labelled with biocytin hydrazide which specifically binds glycoproteins. Biotinylated proteins were purified from cell lysates with immobilized streptavidin. Compared to untreated cells (CTR), FK significantly increased the abundance of AQP2 at the apical plasma membrane, as indicated by the increase in the amount of membrane AQP2 normalized with respect to total AQP2 (FK = 674.2 ± 156.9 vs. CTR = 100 ± 22.48, *n* = 3, ** *p* = 0.006) (densitometry on the right in Figure 7B).Moreover, when FK stimulation was performed in the presence of quinidine, the FK-induced AQP2 membrane accumulation was significantly reduced with respect to FK. Quinidine alone did not affect cell surface expression of AQP2 (Figure 7A,B). 

### 3.9. Chemical K^+^ Gradient Is Required for Fusion of AQP2 Vesicles to the Plasma Membrane: Membrane Fusion Assay

**Figure 8:**
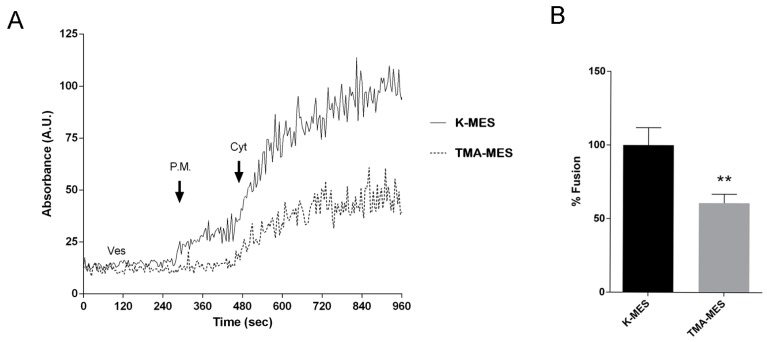
Membrane fusion assay. (**A**) Time course of fluorescence signal of AQP2 vesicles labelled with R18 and incubated with unlabelled plasma membrane after addition of cytosol. Effect of K+ chemical gradient on membrane fusion. Representative curves are shown. (**B**) Statistical analysis of fluorescence signal was calculated as a delta between 120 and 840 s. Statistical analysis was performed by One-way ANOVA. Compared with K-MES (fusion obtained after cytosol addition) the fusion between vesicles and plasma membrane was significantly decreased by about 40% in the absence of K^+^ gradient (TMA-MES). All values are reported as means ± S.E.Ms. ** *p* = 0.007.

To evaluate the relevance of an inwardly chemical K^+^ gradient in the process of AQP2 vesicle fusion to the plasma membrane, we applied a fusion assay, as previously described [32]. This fluorescence technique allows real-time monitoring of fusion between membranes. AQP2 vesicles were isolated and labelled with R18, a fluorescent lipid probe, at self-quenching concentration. As a consequence of fusion with unlabelled plasma membrane, R18 dilution occurs, leading to an increase in the fluorescent emission signal. In the absence of cytosol, virtually no fusion occurs. The fusion occurred upon the addition of cytosol (arrow) indicating that it contains essential factors promoting the fusion (Figure 8A). Compared with control conditions (100 ± 11.54%), substitution of K^+^ in the medium with TMA, preserving the same osmolality, significantly impairs the fusion between vesicles and plasma membranes (39.22 ± 5.83%, *n* = 7, ** *p* = 0.007) supporting the concept that activation of K^+^ channels facilitate the fusion to the plasma membrane and this process requires a K^+^ inwardly chemical gradient. 

## 4. Discussion

In renal collecting duct principal cells, AQP2 vesicles traffic from an intracellular pool and fuse to the apical plasma membrane in response to vasopressin, resulting in water reabsorption from the lumen, a mechanism essential to fine tune water permeability and regulate water homeostasis [25,26,27].

The central hypothesis that is addressed in this study is whether, AQP2 vesicles, an example of non-secretory vesicles committed to insert a channel into the plasma membrane need to swell to facilitate the water channel incorporation into the membrane. Through an interdisciplinary approach, we report here the first evidence that AQP2 vesicle swell in response to activation of a Gi protein-regulated K^+^ channel, that we tentatively identified as Gαi3 and TASK-2 respectively. For this purpose, we have combined high resolution AFM dynamic imaging, a fluorescence-based assay of vesicle volume changes, NMR spectroscopy to measure water self-diffusion coefficient and a fluorimetric cell-free fusion assay.

The first evidence that secretory vesicles undergo an increase in their volume during exocytosis have been provided in different studies published in the early 1990s in mast cells [30,49,50,51]. From these studies, it was concluded that swelling of secretory granules could serve as dual purpose of promoting fusion and rapid release of granule contents. 

Subsequently, other studies have reported evidence that secretory vesicles swell as a consequence of ion and water entry into the vesicle and the resultant pressure generated promotes the discharge of the intravesicular contents to the outside. Of interest, those studies showed that aquaporins, in conjunction with selected ion channels present at the secretory vesicle membrane, regulate the vesicle volume through GTP-binding proteins [5,6,8,52,53,54]. Collectively, these observations elucidated some molecular mechanisms of secretory vesicles swelling supporting the view that this process is required for intravescicular release during secretion.

In our present contribution this concept is revisited, since we show for the first time that AQP2 bearing vesicles subjected to regulated exocytosis upon vasopressin action, swell in response to activation of a Gi protein, Gαi3 that regulates TASK-2 (K^+^ channels) co-expressed with the AQP2, in isolated AQP2 vesicles. 

We provide here evidence that exposure of intact mouse collecting duct MCD4 cells to pertussis toxin (PTX), which ADP-ribosylates and thereby uncouples G proteins of the Gi and Go types from their cognate receptors, inhibited both the cAMP-induced increase in water permeability and the redistribution of AQP2 from an intracellular compartment to the apical membrane, confirming our previous data obtained in rabbit CD8 renal cells [28]. 

Based on these findings, we determined the functional role of Gαi3 in regulating MCD4 water permeability focusing on a putative role in regulation of vesicle swelling as prerequisite for targeting AQP2 to the plasma membrane. We found that isolated AQP2 vesicles from the inner medulla of rat kidney, swell rapidly in response to the Gαi agonist mastoparan which has been demonstrated to activate the GTPase activity of Go/i proteins [19,20,21], suggesting rapid water gating into AQP2 vesicles. The purity of the AQP2 vesicles was determined by the enriched presence of VAMP2 immunoreactivity in isolated vesicles compared to the homogenate. 

Three different biophysical techniques have been applied to demonstrate AQP2 vesicle swelling in response to the Gαi agonist mastoparan. First, a fluorescence-based assay was used, allowing to record the fluorescence signal from vesicles labelled with the R18 probed at self-quenching concentration. R18 probe has been shown to be a reliable tool for investigating of lipid mixing of synaptosomal [55], pancreatic zymogen granules [18] and AQP2 vesicles [32] membranes in vitro. We observed that AQP2 vesicles swell in response to the Gαi agonist mastoparan, within 30 s. While this technique allowed the simultaneous evaluation, in real time, of fluorescence signals from a large number of vesicles plated in multiwells, next, swelling was monitored at single vesicle level at nanoscale resolution using high resolution dynamic imaging with AFM. Imaging before and after treatment with the Gαi agonist mastoparan demonstrated a significant increase both in diameter (from 58.0 ± 5.9 nm to 67.1 ± 7.3 nm, *n* = 16) and height (from 23.7 ± 2.4 nm to 25.4 ± 2.1 nm, *n* = 16) leading to calculated increase in total volume of about 34%. Since 5% is the commonly assumed limit of stretchability, to support nearly 35% volume increase, the presence of actin cytoskeleton may provide additional resistance to swelling. Indeed, the presence of actin inside the vesicles were confirmed in AQP2 vesicles (data not shown). Moreover, we cannot exclude that vesicles may not be unilamellar or may have invaginations since by electron microscopy we could not appreciate these aspects because of not sufficient sensitivity.

While vesicle volume increase has been proposed to be required for fractional release of intravesicular contents from cells during secretion, to our knowledge this is the first evidence that a non-secretory vesicle swell upon Gαi activation associated to the vesicle.

Moreover, a non-invasive technique to investigate water transport across membranes, the NMR-diffusometry, was applied in this study to investigate water diffusion in response to Gi activation with mastoparan. Water self-diffusion coefficient (D) provides information on the dynamic and structure of nano-assemblies in aqueous solutions and was found significantly increased (about 6%, from 1.787 (± 0.002) × 10^−9^ m^2^ s^−1^ to 1.885 (± 0.003) × 10^−9^ m^2^ s^−1^), after AQP2 vesicle exposure to mastoparan. This effect can be due to a double phenomenon: first, taking into account that we are actually observing the mobility of water external to the vesicles, an increase in the average vesicle volume reduces the packing, therefore, water mobility is expected to be less convoluted with respect to that occurring under control conditions, since more empty spaces and less contorted path are created, consequently D increases; second, a component contributing to the increase in D value is the enhanced water movement inside the vesicle, secondary to a gating of a K channel in response to Gi activation, generating an inward osmotic gradient causing rapid water entry into the vesicles mainly through AQP2. In line with these considerations, we found that vesicle pre-treatment with HgCl_2_ reduced the increase in D by about half, that is, about 3%. The obtained data provide a novel, non-invasive and complementary way to measure water exchange in equilibrium exchange conditions. A very recent work highlighted the utility of the relaxometry (T1 and T2) and diffusometry (PFG) NMR spectroscopy to investigate the transport properties and the diffusional exchange across cell membrane in yeast cells with and without aquaporins [56]. 

It is known that water can move across biological membranes through diffusion, a relatively slow temperature-sensitive process and through aquaporins, allowing a more rapid, osmotically driven passage of water. The AQP-mediated water transport is sensitive to mercuric chloride which binds to sulfhydryl groups (cysteine residues) on water channels and inhibits their function. Based on AFM vesicle imaging, mercurial chloride (HgCl_2_) prevented vesicle swelling causing instead a vesicle volume decrease. This may be explained with a possible reduction in the intravesicular osmolyte concentration associated with HgCl_2_ effects on other transporters sensitive to HgCl_2_.

While isolated AQP2 vesicles used in this study were found to express negligible contamination of AQP1, AQP3 and AQP4, we have no direct prove of AQP2-mediated water entry since HgCl_2_ does not represent a specific AQP inhibitor. In principle, the presence of an AQP mediating water influx into the vesicle is expected to speed the vesicle swelling, which might be fundamental in regulated exocytosis requiring a very rapid response to the hormonal stimulus (vasopressin). In line with this hypothesis, all the known examples of secretory vesicle swelling in response to a given stimulus, have been suggested to involve an aquaporin like AQP1 [4], AQP5 [22]; and AQP6 (synaptic vesicles [23]). Nevertheless, as a general mechanism, AQPs may not be essential for vesicle fusion to the plasma membrane since the lipid membrane is permeable to water. Indeed, in general, AQPs knockout mice do not display impaired synaptic or endocrine functions [57]. Due to the wide variety of effects of HgCl_2_ on other membrane transporters, future experiments using AQP2 vesicles isolated from AQP2 KO mice will provide a definitive answer for the requirement of AQP2 mediated water entry for vesicle fusion to the plasma membrane.

Water flow is driven by a concentration gradient to equilibrate the osmotic concentration on both sides of the biological membranes. In this regard, we report here that vesicle swelling in response to activation of a Gαi associated to the membrane, is likely mediated by gating of K^+^ channel co-expressed with Gαi in vesicles and tentatively identified as TASK-2. TASK-2 is a pH sensitive K^+^ channel cloned from human kidney and expressed in cortical distal tubule and collecting duct [44]. TASK-2 is modulated by heterotrimeric G protein and have been proposed to be involved in regulatory volume decrease (RVD), a process in which cells swollen in response to hypotonicity recover their volume by activation of K^+^ and Cl^−^ channels followed by KCl efflux and osmotically obliged water [58].

**Figure 9:**
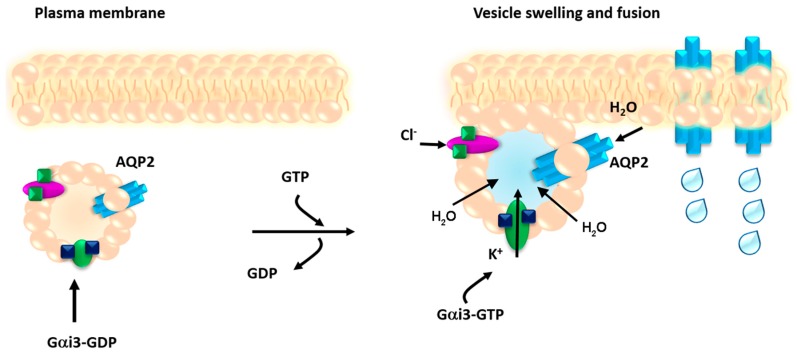
Schematic hypothetical model of AQP2 vesicle swelling. Activation of Gαi3 protein results in the opening of K^+^ channels (TASK-2), causing K^+^ entry into the vesicle due to a positive inward chemical gradient. The positive charge of K^+^ is balanced by Cl^−^ entry through ClC-K channels also identified in isolated AQP2 vesicles. This initially created osmotic gradient draws water into the vesicles resulting in vesicles swelling.

By mass spectrometry, TASK-2, which is G protein regulated channel [46], has been predicted to be expressed in immunoisolated vesicles [43]. TASK-2 currents are blocked by quinidine (but not by the other classical K^+^ channel blockers) [59]. Notably, we found that inhibition of this channel with quinidine prevented the effect of Gαi on vesicle swelling, AQP2 translocation and the increase in osmotic water permeability in intact renal MCD4 cells (Figure 6A,B) pointing to a crucial role of this K^+^ channel in generating an inward osmotic gradient secondary to K^+^ entry into the vesicle (see model Figure 9). 

Interestingly, TASK-2 is very sensitive to pH and 90% of its current is recorded at pH 7.7 whereas only 10% at pH 6.7 [59]. Of note, vasopressin binding to its V2 receptor induces intracellular alkalinisation via NHE1 activation [60] and this effect might have physiological relevance in triggering TASK-2 activation to promote AQP2 bearing vesicle swelling and fusion into the apical membrane. In line with these data, a recent study showing that dDAVP-induced phosphorylation and apical targeting of AQP2 are attenuated in IMCD cells under acidic extracellular pH [61]. 

In mammalian cells, the cytosolic K^+^ concentration is elevated (around 140 mM) and is likely that, upon TASK-2 gating by Gi activation, K^+^ entry driven by its chemical gradient, generates an osmotic gradient causing water entry into the vesicle resulting in vesicle swelling. Indeed, for the first time we have been able to measure the intravesicular K^+^ concentration in AQP2 vesicles and found to be around 5mM providing a strong rationale that vesicle swelling may occur even in living cells triggered by TASK-2 gating in response to vasopressin stimulation.

This study does not address the issue of what is the ion that balance the entry of a positive charge. A possible candidate is Cl^−^, however while the Cl channel ClC-K was found to be expressed in AQP2 vesicles, blocking the Cl^−^ channel with the generic inhibitor DIDS, had no apparent effect on Gi-activated vesicle swelling.

There are several questions that arise in this context: a. in response to an external stimulus in live cells, when does vesicle swelling occur? b. why this process would facilitate the insertion of the AQP2 water channel in the plasma membrane? c. what is the upstream signal linked to vasopressin stimulation causing Gi3-regulated K^+^ channel gating? 

The present contribution gives some input to these issues. By employing a fusion assay, we demonstrated that, in a cell free system, the fusion between isolated vesicles and plasma membrane was significantly inhibited by substitution of K^+^ in the medium with TMA, supporting the concept that activation of K^+^ channels facilitate the fusion to the plasma membrane and this process requires a K^+^ inwardly chemical gradient.

Concerning the second point it can be speculated that upon swelling vesicle membrane bilayer gets thinner and the negative area density of the lipid head declines facilitating the interaction with the plasma membrane. Early data using small unilamellar vesicles would support this view demonstrating that vesicle fusion process is greatly facilitated by the increment of tension owing to the substantial reduction in the time periods for adhesion and hemifusion processes [62]. Third, the upstream signal(s) linked to vasopressin stimulation causing Gi3-regulated K^+^ channel gating have to be defined. Besides vasopressin induced cytosol alkalinisation which may facilitate vesicle K^+^ channel opening, the possibility that a vesicle resident GPCR can activate the heterotrimeric Gi is intriguing and deserves future investigations. In this view, the presence of functional beta adrenergic receptors at the synaptic vesicle membrane regulating vesicle swelling has been demonstrated [54].

In summary, we show here that Gi modulation of potassium channel TASK-2 mediates vesicle osmotic swelling required for AQP2 vesicle fusion to the plasma membrane in response to cAMP-elevating agents. While our data represent the first example that vesicle swelling is required for fusion of a non-secretory vesicle committed to insert a channel into the plasma membrane, the generalization to other non-secretory vesicles needs further investigation.
